# Predicting Cerebral Hyperperfusion Syndrome Following Superficial Temporal Artery to Middle Cerebral Artery Bypass based on Intraoperative Perfusion-Weighted Magnetic Resonance Imaging

**DOI:** 10.1038/srep14140

**Published:** 2015-09-14

**Authors:** Defeng Wang, Fengping Zhu, Ka Ming Fung, Wei Zhu, Yishan Luo, Winnie Chiu Wing Chu, Vincent Chung Tong Mok, Jinsong Wu, Lin Shi, Anil T. Ahuja, Ying Mao

**Affiliations:** 1 Department of Imaging and Interventional Radiology, The Chinese University of Hong Kong, Shatin, New Territories, Hong Kong, China; 2 Research Center for Medical Image Computing, Department of Imaging and Interventional Radiology, The Chinese University of Hong Kong, Shatin, Hong Kong SAR, China; 3 Department of Neurosurgery, Huashan Hospital, Fudan University, Shanghai, China; 4 Shenzhen Research Institute, The Chinese University of Hong Kong, Shenzhen, China; 5 Department of Biomedical Engineering and Shun Hing Institute of Advanced Engineering, The Chinese University of Hong Kong, Shatin, Hong Kong SAR, China; 6 Australian School of Advanced Medicine, Macquarie University, Sydney, Australia; 7 Department of Medicine and Therapeutics, The Chinese University of Hong Kong, Shatin, Hong Kong SAR, China; 8 Lui Che Woo Institute of Innovative Medicine, The Chinese University of Hong Kong, Shatin, Hong Kong SAR, China

## Abstract

Moyamoya disease leads to the formation of stenosis in the cerebrovasculature. A superficial temporal artery to middle cerebral artery (STA-MCA) bypass is an effective treatment for the disease, yet it is usually associated with postoperative cerebral hyperperfusion syndrome (CHS). This study aimed to evaluate cerebral hemodynamic changes immediately after surgery and assess whether a semiquantitative analysis of an intraoperative magnetic resonance perfusion-weighted image (PWI) is useful for predicting postoperative CHS. Fourteen patients who underwent the STA-MCA bypass surgery were included in this study. An atlas-based registration method was employed for studying hemodynamics in different cerebral regions. Pre- versus intraoperative and group-wise comparisons were conducted to evaluate the hemodynamic changes. A postoperative increase in relative cerebral blood flow (CBF) at the terminal MCA territory (*P *= 0.035) and drop in relative mean-time-transit at the central MCA territory (*P *= 0.012) were observed in all patients. However, a significant raise in the increasing ratio of relative-CBF at the terminal MCA territory was only found in CHS patients (*P *= 0.023). The cerebrovascular changes of the patients after revascularization treatment were confirmed. Intraoperative PWI might be helpful in predicting the change in relative-CBF at MCA terminal territory which might indicate a risk of CHS.

Moyamoya disease (MMD) is characterized by stenosis or occlusion at the terminal portions of the internal carotid artery (ICA) or the proximal areas of the anterior cerebral arteries (ACA) or middle cerebral arteries (MCA)^1^. It is accompanied by a formation of fine vascular networks at the base of the brain, which looks like “a puff of smoke” in an angiogram. Although randomized controlled trials have not been performed, there have been strong indications from observational studies that a direct superficial temporal artery to middle cerebral artery (STA-MCA) bypass with or without indirect revascularization could reduce the risk of cerebral ischemic or hemorrhagic stroke through flow augmentation and collateralization reduction for patients with MMD^1^.

STA-MCA anastomosis was traditionally considered to provide a low-flow bypass for MMD patients. However, it is associated with postoperative symptomatic cerebral hyperperfusion syndrome (CHS) with a high incidence of 16.7%–71.4%, which might lead to severe neurological deficits^2^,^3^. CHS is a group of symptoms after revascularization with severe headache, epilepsy, disturbance of consciousness, and focal neurological deficits as the main clinical manifestations^4^. Postoperative CHS results from the quick restoration of blood flow to a chronically hypoperfused brain and potentially leads to brain edema or intracerebral hemorrhage^5^. Although the mechanisms underlying CHS remain unclear, the impaired autoregulation, endothelial dysfunction mediated by free radicals, and breakdown of the baroreceptor reflex might contribute to its pathophysiology^5^,^6^. Detailed investigation of cerebral hemodynamic changes following STA-MCA bypass at an early stage is important to predict and avoid the occurrence of postoperative CHS.

Perfusion-weighted magnetic resonance imaging (PWI) is strongly correlated with positron emission tomography (PET) in studying cerebral hemodynamics. As a noninvasive measurement, PWI reflects hemodynamic information of the brain tissue and subsequently obtains quantitative information through calculations of the mathematical models, including cerebral blood flow (CBF), cerebral blood volume (CBV), and mean transit time (MTT)^7^. Therefore, this technique is theoretically an effective tool in assessing the surgical effect and predicting postoperative CHS following cerebral revascularization surgery.

Currently, the standardized clinical definition of CHS is not yet established, and the advanced neuroimaging methods may be less fully utilized. The definitions of CHS used in the literature include clinical symptoms unrelated to cerebral ischemia. Some combined with the imaging criteria that CBF increases greater than 100% over the baseline of the contralateral MCA territory after the procedure are identified either by perfusion imaging, PET or single-photon emission computed tomography (SPECT) imaging^8^. However, the imaging definition remains variable. The symptoms of CHS may occur without any increase in CBV or CBF. Conversely, patients with more than 100% increase in CBF after the procedures might not develop clinical CHS^5^,^9^. Furthermore, these neuroimaging modalities are either unreliable, heavily operator dependent or unobtainable in a significant proportion of patients^10^. To the best of our knowledge, intraoperative PWI has not been reported in the usage of monitoring STA-MCA bypass surgery. The aim of this study is to evaluate the application value of intraoperative PWI on bypass surgery and to determine its role in predicting the surgical effect and postoperative CHS.

## Methods

### Subjects

This study included 14 patients who underwent STA-MCA anastomosis and encephaloduromyosynangiosis (EDMS) surgery in the 3.0T intraoperative magnetic resonance imaging (iMRI) operating suites at the Huashan Hospital. MMD was diagnosed according to the criteria of the Research Committee on Spontaneous Occlusion of the Circle of Willis (Moyamoya Disease) of the Ministry of Health and Welfare, Japan^11^. There were six males and eight females diagnosed of MMD with a mean age of 35.3 ± 5.1 years, ranging from 25 to 46 years. The study was approved by the local ethical committee of Huashan Hospital. Written informed consent was obtained from each patient. All experiments were performed in accordance with relevant guidelines and regulations set out by the ethical committee.

### Surgical procedures and diagnosis of postoperative CHS

Surgical intervention for MMD was indicated after comprehensively evaluating the digital subtraction angiography (DSA), clinical manifestations and brain perfusion or metabolic findings at the Huashan Hospital. The detailed surgical indications were described in Xu and *et al*.^12^. For each patient, standard STA-MCA anastomosis was performed. Briefly, a modified pterional approach was adopted. The frontal and/or parietal branch of STA was carefully dissociated from the scalp and then pulled through the temporal muscle to reach the surface MCA. STA was anastomosed with the cortical branch of MCA in an end-to-side fashion using a single 10-0 nylon atraumatic suture. Pressurized normal saline containing heparin was used to relieve spasm of the artery. In this study, CHS was defined as the postoperative development of a severe headache, new neurological deficits without cerebral infarction, seizure, or intracerebral hemorrhage.

### Imaging parameters

iMRI scans were performed with a mobile MRI system (IMRIS, Innovative Magnetic Resonance Imaging Systems, Inc., Winnipeg, MB, Canada) interfaced to a 3.0T MR system console (Verio, Siemens Medical Systems, Erlangen, Germany) after the STA-MCA anastomosis within one hour for all patients. Time-of-flight (TOF) magnetic resonance angiography (MRA) scans (4 slabs, 40 slices/slab; repetition time, 22 ms; echo time, 4.2 ms; flip angle, 18; matrix size, 365 × 384; slice thickness, 0.5 mm; field of view, 181 × 200 mm^2^) were obtained to reconstruct the 3-dimensional brain template.

PWI was performed based on the enhanced fat-suppressed T2 single-shot echo-planar imaging sequence with the following acquisition parameters: repetition time, 1500 ms; echo time, 30 ms; filed of view, 230 × 230 mm^2^; matrix size, 128 × 128; number of layers, 19; slice thickness, 4 mm; interval, 1.2 mm; flip angle, 90°. The contrast agent, gadolinium-diethylenetriamine pentaacetic acid (Gd-DTPA) (Magnevist, Bayer, Berlin, Germany), was administered into an antecubital vein using a power injector (the OptiStar LE Mallinckrodt, Liebel-Flarsheim Company, USA) with a dosage of 0.2 mmol/kg and an injection rate of 4 ml/s.

### PWI post-processing

PWIs were post-processed with a software called Perfusion Mismatch Analyzer (PMA) (Ver.3.4.0.6, ASIST, Japan). The software computed CBF, CBV, and MTT by estimating the contrast concentration using deconvolution by numerical methods. The concentration of the contrast at a particular voxel in the brain, C_v_(t) was obtained by the time series PWI. C_v_(t) was related to the tissue flow, **F**, the fraction of contrast remaining in that voxel, or the residue function, R(t), and the arterial input function, AIF(t), by the equation^13^:


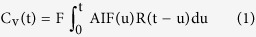


C_v_(t) was obtained by measuring the contrast concentration from PWI. The algorithm searched the AIF voxels in the whole image according to the histograms of peak contrast concentration, time-to-peak (TTP) and MTT^14^,^15^,^16^. By varying the threshold of TTP and MTT, the potential AIF voxels could be identified while adjusting the threshold of peak concentration could determine the number of AIF voxels selected automatically^16^. A manual checking was conducted in each calculation to ensure the pixels selected were located in areas adjacent to the MCA territories^17^. Besides, an assumption on F as a constant value was made.

In each voxel, PMA calculated R(t) by deconvolution using singular value decomposition (SVD)^18^. It was done by discretizing Eq. (1):


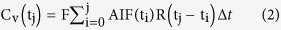


where Δt is the sampling time interval. Then, Eq. (2) could be further formulated as an inverse matrix problem and could be solved by SVD to obtain R(t). Practically, AIF(t) can lag C_v_(t) by a time delay in diseased regions which leads to errors in R(t) estimation. This could be tackled by replacing discretized AIF(t) with a block-circulant matrix which has been shown to be equivalent to linear convolution with time aliasing^19^.

The values of R(t) obtained were then plotted against time and the three perfusion parameters were calculated. For each voxel, CBF was evaluated by the peak of R(t) and CBV was determined by the area under the curve of R(t). By the central volume principle, MTT was the ratio of CBV to CBF^20^.

### Definition of atlas

**Figure 1:**
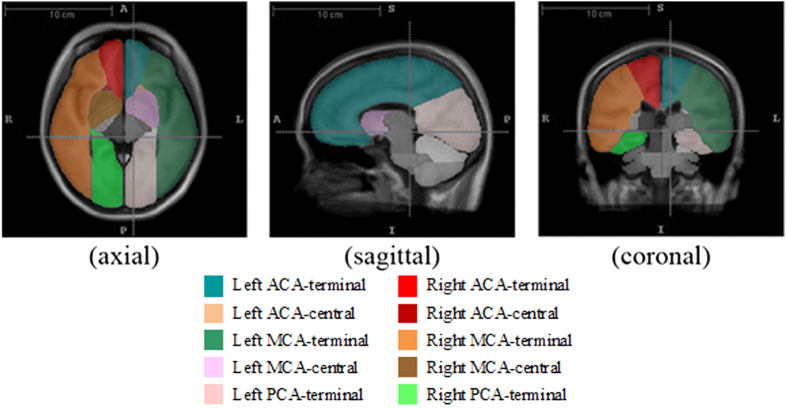
Screenshots shows the axial, sagittal and coronal views and of the brain template and atlas used in this study.

Using the MRA data, a template for the cohort was constructed using group-wise registration in ANTs toolbox (http://www.picsl.upenn.edu/ANTS). With an existing Chinese MRI brain template and an atlas for arterial territories of human brain as proposed by Tatu *et al*.^21^, the atlas for the template in this study was generated using registration-based segmentation techniques. The atlas indicated various arterial regions separated into left and right sides, including the terminal and central brain regions with blood supply from ACA, MCA, and posterior cerebral arteries (PCA) as well as other arterial supply territories. Since MMD is characterized by progressive occlusions at ACA and MCA, the analysis focused on the central and terminal territories of ACA & MCA, and the terminal territory of PCA (Fig. 1).

After the brain template space and its atlas were created, all pre- and intraoperative PWIs were mapped onto the template space by the coregister function of SPM8 (Wellcome Department of Imaging Neuroscience, London, United Kingdom). In each brain segment, the mean values of the three perfusion parameters were computed for further analysis.

### Statistical analysis

With the averaged parameters, a two-tailed paired Student’s t-test was used to compare the pre- and intraoperative perfusion parameters in the ACA, MCA, and PCA territories. And independent sample t-test was used for across-group comparisons. To minimize the symmetric error of left and right hemispheres, we used relative values of the parameters for comparison (i.e., relative-CBF, relative-CBV, and relative-MTT). The relative values were the ratio of parameter value of the surgical side to that on the contralateral side^22^. A significant level of 0.05 was chosen to verify if the changes in the perfusion parameters were statistically significant. The Benjamini-Hochberg Procedure was carried out for multiple comparison correction of the test results with a predefined false discovery rate of 0.2. Pre- and intraoperative comparisons were conducted for all patients and repeated for CHS and non-CHS groups separately.

## Results

Intraoperative MRA and PWI were successfully conducted in each patient. The patency of the bypass graft was evaluated during surgery, using intraoperative MRA and a Doppler flow meter. Postoperative DSA, MRA, and/or computed tomography angiography (CTA) also confirmed the patency of bypass graft in all 14 patients. There was no hemorrhagic or ischemic infarction during the operation. The numerical results are summarized in Table 1, Table 2, and Table 3.

### Patients and clinical outcome

Five cases (35.7%) suffered from postoperative complications, including seizure attacks in two cases, severe headache in one case, aphasia in one case, and transient motor deficit in one case. All fourteen patients recovered well without neurological deficits within two weeks. For further PWI parameter analysis, the case series were divided into two groups. One was the CHS group, in which patients manifested postoperative complications caused by suspected CHS after surgery (5 cases), and the other group was the non-CHS group in which patients had no complications after surgery (9 cases).

### Preoperative versus intraoperative comparisons for all patients

STA-MCA bypass surgeries effectively improved cerebral blood flow at the surgical side. The mean relative-CBF at the terminal MCA territory increased from 0.98 ± 0.10 to 1.03 ± 0.14 after bypass on the surgical side of all patients (*P *= 0.035, versus preoperative data, insignificant after correction). No significant increase was observed for relative-CBF at the central territory of ACA, PCA and MCA. Relative-MTT values at all other regions were insignificant (*P *> 0.05 for all, versus preoperative data). For all patients, the changes of relative-CBV were insignificant at all regions.

### In-group preoperative versus intraoperative comparisons

For the CHS group, the average relative-CBF rose from 1.03 ± 0.14 to 1.17 ± 0.13 at the terminal MCA territory (*P *= 0.011). However, no significant difference for relative-CBF at other regions was observed. There were trends showing increases in relative-MTT at the terminal MCA and PCA territories, but the p-values were insignificant. At PCA, the CHS group had an elevated mean relative-CBV from 0.84 ± 0.10 to 1.00 ± 0.12 (*P *= 0.005), while there were no significant changes at the other territories.

For those without CHS, there were no significant changes to both relative-CBF and relative-MTT in all regions. The relative-CBV for this group changed from 1.04 ± 0.10 to 0.95 ± 0.17 (*P *= 0.015) at PCA and was without changes in other regions.

### Across-group preoperative versus intraoperative comparisons

**Figure 2:**
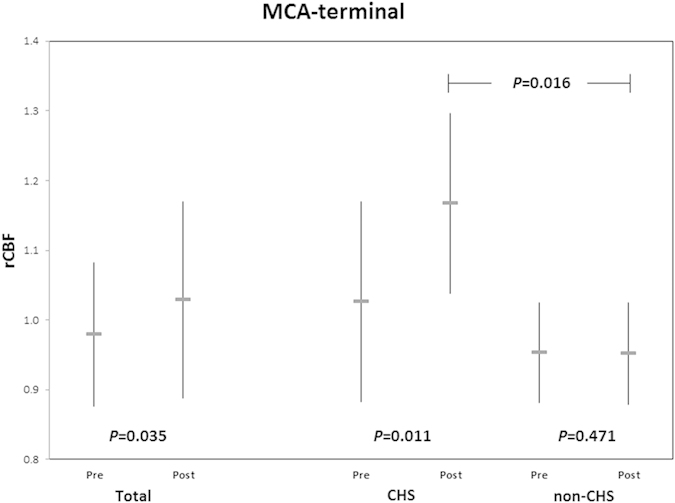
Graph shows mean ± SD of pre- and intraoperative relative-CBF values in terminal MCA territory of CHS and non-CHS patients.

At the MCA-terminal region, the intraoperative relative-CBF values of patients with CHS and those without CHS were significantly different (1.17 versus 0.95 respectively, *P *= 0.016) (Fig. 2). The percentage increase of intraoperative relative-CBF of the CHS group was also significantly larger than that for the non-CHS group (14.3% versus 0.0% respectively, *P = *0.023).

At the PCA territory, the preoperative value and percentage change of intraoperative relative-CBV for the CHS group were significantly different from that of the non-CHS group (0.84 vs 1.04 with *P *= 0.010 and 19.8% vs −8.9% with *P *= 0.001 respectively). No other significant difference between the two groups was observed in our results.

### Illustrative case

**Figure 3:**
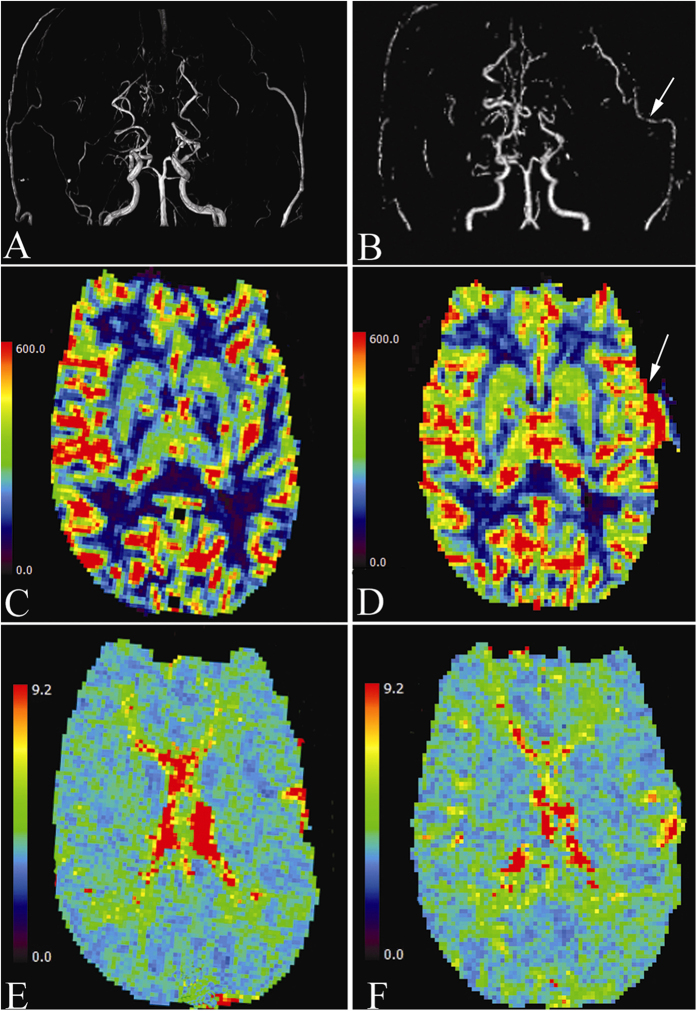
(**A**) Preoperative MRA showing bilateral occlusion of the proximal ICA and Moyamoya change of intracranial vessels. (**B**) Intraoperative MRA showing the patency of left STA-MCA bypass graft (arrow). (**C**) Preoperative PWI demonstrating diminished CBF of the left hemisphere. (**D**) Intraoperative PWI demonstrating CBF of the left hemisphere markedly increased, mainly at the terminal MCA territory (arrow). (**E**) Preoperative PWI demonstrating prolonged MTT of the left hemisphere, especially at the terminal MCA territory. (**F**) Intraoperative PWI indicating the prolonged MTT of the left hemisphere didn’t change obviously.

A 36-year-old man presented transient weakness of the right extremities, and was diagnosed as having MMD. Preoperative PWI revealed decreased CBF and increased MTT in the left MCA and ACA territories, suggesting a severe hemodynamic compromise. Left STA-MCA anastomosis plus EDMS surgery was successfully performed. Intraoperative MRA confirmed the patency of the bypass graft. Intraoperative PWI revealed the relative-CBF at the MCA-terminal territory markedly increased from 0.95 to 1.18 at a rate of 24.3%, however, relative-MTT at the terminal MCA territory just decreased from 0.97 to 0.96 at a rate of 1.1% (Fig. 3). The patient developed focal seizure attacks (2–3 times per day) 1 day after surgery. After strict blood pressure control, administering antiepileptic medications, seizure attacks were well controlled 4 days later. He developed no neurological dysfunction during the perioperative period.

## Discussion

Awareness of CHS is one of the keys to improved bypass procedures. The earlier the potential risks of CHS are identified, the lower the incidence rate of postoperative complications. This study demonstrated that semiquantitative analysis of intraoperative PWI might be an effective method for predicting postoperative CHS after STA-MCA anastomosis.

iMRI was widely used to provide real-time images for neuronavigation in brain tumor surgery^23^. However, it is rarely reported to be used in the neurovascular surgery. It was proven to be helpful in preventing insidious or early complications during operation, such as cerebral ischemia and hemorrhage^24^. It may be too early to comment upon the clinical superiority of iMRI over postoperative MRI in the detection of cerebral hyperperfusion after considering the high-cost iMRI. But, several potential benefits could be observed. In previous studies, postoperative MRI was usually performed when hyperperfusion symptoms occurred or within one week after the bypass procedure^3^,^25^. Intraoperative PWI could provide earlier information of hemodynamic changes immediately after the reestablishment of blood flow compared with postoperative MRI. Once patients with a high risk of CHS were identified, intensive care and earlier treatment could be performed. Furthermore, the patency of bypass may be assessed intraoperatively. If an abnormal increase in the relative-CBF was to be observed using intraoperative PWI and MRA, the surgeons might revise or optimize the revascularization procedures.

Another highlight of this study is the application of an atlas-based method for cerebral hemodynamic analysis. In the previous MMD research, scholars mainly carried out such analysis using a manual selection of a region of interest^14^,^26^. The atlas-based method considered the brain in several sections according to a well-established brain template automatically^27^,^28^,^29^,^30^ and minimized the potential selective errors. Atlas-based methods provide a holistic picture of the brain hemodynamics. With the perfusion parameters, the hemodynamics of brains could be visualized like Fig. 3 introduced with the illustrative case.

This study preliminarily quantified intraoperative cerebral hemodynamic changes after STA-MCA bypass procedures and tried to stratify patients at risk for postsurgical CHS. Intraoperative real-time analysis provided some different results compared with previous studies using postoperative PWI. When analyzing data in the whole case series, statistically significant increases in the relative-CBF at the terminal MCA territory after surgery were found. This implied that the blood flow of the surgical cortex was immediately augmented after bypass procedures. The intraoperative relative-CBF value at the terminal MCA territory might be a sensitive parameter reflecting the bypass effect^22^,^31^,^32^. However, relative-CBF values at other areas of arterial territories revealed no significant changes. It indicated that the bypass approach is important to surgical effect and the decision on the specific surgical procedure should be based on a detailed hemodynamic analysis.

More importantly, for patients with CHS, increases in relative-CBF at the MCA-terminal regions have been found. Relative-CBF at the terminal MCA territory increased for about 14.3% of the preoperative values in five patients who developed postoperative CHS on PWI scans immediately after surgery. The value was significantly different from the non-CHS group whose average preoperative-intraoperative relative-CBF change was about 0.0%. So, the immediate great increases of relative-CBF at the terminal MCA territory might be of prognostic value for postoperative CHS which is categorized by a generally agreed upon relative-CBF increment of more than 100% of the preoperative value^33^. The cutoff value of the variation rate of the relative-CBF at the terminal MCA territory for predicting postoperative CHS might be made in the future.

Among the fourteen patients, CHS was found in 5 of them after revascularization surgery. Referring to some similar studies, it was expected that there would be significant increases in their CBF and CBV and delays in MTT^22^,^30^,^32^,^34^. A possible cause of the discrepancies could be the short time interval for capturing the intraoperative images after bypass surgeries and the cerebral vascular networks may not be fully developed in such a short time.

Other sources of error in the perfusion parameters change were the image quality and the deconvolution method used by PMA. Deconvolution is highly sensitive to image noise which could lead to great errors in the determination of the residue function and thus the perfusion parameters. Besides, the arterial input function was automatically detected by the software. It might potentially contribute the errors of the calculated hemodynamic changes after surgery.

The number of subjects was limited in the present study. An extended research with a larger set of cases will be needed to confirm the result. Furthermore, follow-up scans and examinations should be performed to estimate the clinical effectiveness of surgery in the long run.

In conclusion, this study demonstrated that semiquantitative analysis of atlas-based intraoperative PWI could be an effective method for assessing surgical effectiveness and predicting postoperative CHS after STA-MCA anastomosis. Changes in the cerebrovasculature of MMD patients after revascularization treatments were confirmed. Immediate increases of relative-CBF at the terminal MCA territory during operation might be regarded as a sensitive indicator for the occurrence of postoperative CHS.

## Additional Information

**How to cite this article**: Wang, D. *et al*. Predicting Cerebral Hyperperfusion Syndrome Following Superficial Temporal Artery to Middle Cerebral Artery Bypass based on Intraoperative Perfusion-Weighted Magnetic Resonance Imaging. *Sci. Rep*. **5**, 14140; doi: 10.1038/srep14140 (2015).

## Figures and Tables

**Table 1:** Summary of mean relative cerebral blood flow (relative-CBF).

	All Patients (n = 14)	CHS Group (n = 5)	Non-CHS Group (n = 9)	P-value (CHS vs non-CHS)
ACA-terminal				
- Pre	0.93	0.90	0.95	0.590
- Intra	0.92	0.93	0.91	0.818
- %increase	–0.2%	5.0%	–3.2%	0.162
P-value (Pre vs Intra)	0.338	0.210	0.110	
ACA-central				
- Pre	0.85	0.77	0.90	0.567
- IntraIntra	1.01	0.99	1.03	0.862
- %increase	32.7%	58.6%	18.4%	0.518
P-value (Pre vs Intra)	0.047	0.169	0.098	
MCA-terminal				
- Pre	0.98	1.03	0.95	0.333
- Intra	1.03	1.17	0.95	0.016*
- %increase	5.1%	14.3%	0.0%	0.023*
P-value (Pre vs Intra)	0.035	0.011*	0.471	
MCA-central				
- Pre	0.96	1.07	0.90	0.386
- Intra	0.98	1.10	0.91	0.357
- %increase	2.3%	4.3%	1.2%	0.721
P-value (Pre vs Intra)	0.266	0.347	0.326	
PCA-terminal				
- Pre	1.01	0.92	1.06	0.268
- Intra	0.99	0.92	1.02	0.303
- %increase	−1.2%	3.4%	−3.8%	0.338
P-value (Pre vs Intra)	0.241	0.452	0.146	

^*^significant P-values after multiple comparison correction.

**Table 2:** Summary of mean relative cerebral blood volume (relative-CBV).

	All Patients (n = 14)	CHS Group (n = 5)	Non-CHS Group (n = 9)	P-value (CHS vs non-CHS)
ACA-terminal				
- Pre	0.97	0.98	0.97	0.893
- Intra	0.94	0.94	0.94	0.986
- %increase	−2.3%	−3.3%	−1.8%	0.863
P-value (Pre vs Intra)	0.240	0.296	0.336	
ACA-central				
- Pre	0.88	0.88	0.88	0.998
- Intra	1.06	1.02	1.09	0.777
- %increase	33.2%	41.7%	28.5%	0.802
P-value (Pre vs Intra)	0.152	0.293	0.206	
MCA-terminal				
- Pre	1.04	1.12	0.99	0.503
- Intra	1.05	1.32	0.90	0.152
- %increase	0.7%	17.7%	−8.8%	0.083
P-value (Pre vs Intra)	0.436	0.085	0.127	
MCA-central				
- Pre	0.99	1.09	0.93	0.417
- Intra	0.99	1.15	0.90	0.275
- %increase	1.5%	8.1%	−2.2%	0.460
P-value (Pre vs Intra)	0.493	0.308	0.318	
PCA-terminal				
- Pre	0.97	0.84	1.04	0.010*
- Intra	0.97	1.00	0.95	0.472
- %increase	1.4%	19.8%	–8.9%	0.001*
P-value (Pre vs Intra)	0.484	0.005*	0.015*	

^*^significant P-values after multiple comparison correction.

**Table 3:** Summary of mean relative mean transit time (relative-MTT).

	All Patients (n = 14)	CHS Group (n = 5)	Non-CHS Group (n = 9)	P-value (CHS vs non-CHS)
ACA-terminal				
- Pre	1.09	1.19	1.04	0.534
- Intra	1.03	1.03	1.04	0.954
- %increase	−2.2%	−8.2%	1.1%	0.347
P-value (Pre vs Intra)	0.190	0.190	0.479	
ACA-central				
- Pre	1.04	1.24	0.93	0.088
- Intra	0.98	1.02	0.96	0.612
- %increase	−3.9%	−14.7%	2.1%	0.226
P-value (Pre vs Intra)	0.202	0.103	0.366	
MCA-terminal				
- Pre	1.03	1.06	1.01	0.703
- Intra	1.00	1.07	0.97	0.428
- %increase	−2.0%	2.1%	−4.3%	0.520
P-value (Pre vs Intra)	0.244	0.476	0.104	
MCA-central				
- Pre	1.07	1.05	1.08	0.563
- Intra	0.97	0.99	0.96	0.583
- %increase	−8.9%	−4.8%	−11.1%	0.340
P-value (Pre vs Intra)	0.012	0.107	0.030	
PCA-terminal				
- Pre	1.00	1.05	0.97	0.690
- Intra	1.05	1.20	0.97	0.312
- %increase	9.1%	24.5%	0.6%	0.332
P-value (Pre vs Intra)	0.112	0.115	0.459	

*significant P-values after multiple comparison correction.
